# A multi-centre randomised trial to compare the effectiveness of geriatrician-led admission avoidance hospital at home versus inpatient admission

**DOI:** 10.1186/s13063-017-2214-y

**Published:** 2017-10-23

**Authors:** Sasha Shepperd, Andrea Cradduck-Bamford, Chris Butler, Graham Ellis, Mary Godfrey, Alastair Gray, Anthony Hemsley, Pradeep Khanna, Peter Langhorne, Patricia McCaffrey, Lubena Mirza, Maj Pushpangadan, Scott Ramsay, Rebekah Schiff, David Stott, John Young, Ly-Mee Yu

**Affiliations:** 1 Nuffield Department of Population Health, University of Oxford, Richard Doll Building, Old Road Campus, Oxford, OX3 7LF UK; 2 Nuffield Department of Primary Care Health Sciences, University of Oxford and Cardiff University, Cardiff, UK; 3 Monklands Hospital, NHS Lanarkshire, Glasgow, UK; 4 Institute of Health Sciences, University of Leeds, Leeds, UK; 5 Royal Devon and Exeter NHS Foundation Trust, Exeter, Devon UK; 6 Aneurin Bevan University Health Board, Newport, South Wales UK; 7 Institute of Cardiovascular and Medical Sciences, University of Glasgow, Glasgow, UK; 8 Southern Health and Social Care Trust, Craigavon, UK; 9 Academic Unit of Elderly Care and Rehabilitation, University of Leeds, Leeds, UK; 10 St John’s Hospital, NHS Lothian, Livingstone, UK; 11 Guy’s and St Thomas’ Hospital, London, UK; 12 Nuffield Department of Primary Care Health Sciences, University of Oxford, Oxford, UK

## Abstract

**Background:**

There is concern that existing models of acute hospital care will become unworkable as the health service admits an increasing number of frail older people with complex health needs, and that there is inadequate evidence to guide the planning of acute hospital level services. We aim to evaluate whether geriatrician-led admission avoidance to hospital at home is an effective alternative to hospital admission.

**Methods/Design:**

We are conducting a multi-site randomised open trial of geriatrician-led admission avoidance hospital at home, compared with admission to hospital. We are recruiting older people with markers of frailty or prior dependence who have been referred to admission avoidance hospital at home for an acute medical event. This includes patients presenting with delirium, functional decline, dependence, falls, immobility or a background of dementia presenting with physical disease. Participants are randomised using a computerised random number generator to geriatrician-led admission avoidance hospital at home or a control group of inpatient admission in a 2:1 ratio in favour of the intervention. The primary endpoint ‘living at home’ (the inverse of death or living in a residential care setting) is measured at 6 months follow-up, and we also collect data on this outcome at 12 months. Secondary outcomes include the incidence of delirium, mortality, new long-term residential care, cognitive impairment, activities of daily living, quality of life and quality-adjusted survival, length of stay, readmission or transfer to hospital. We will conduct a parallel economic evaluation, and a process evaluation that includes an interview study to explore the experiences of patients and carers.

**Discussion:**

Health systems around the world are examining how to provide acute hospital-level care to older adults in greater numbers with a fixed or shrinking hospital resource. This trial is the first large multi-site randomised trial of geriatrician-led admission avoidance hospital at home, and will provide evidence on alternative models of healthcare for older people who require hospital admission.

**Trial registration:**

ISRCTN60477865: Registered on 10 March 2014. Trial Sponsor: University of Oxford. Version 3.1, 14/06/2016.

**Electronic supplementary material:**

The online version of this article (doi:10.1186/s13063-017-2214-y) contains supplementary material, which is available to authorized users.

## Background

Older people are being admitted to hospital as an emergency in increasing numbers [1]. From a system perspective this trend is not sustainable, and from a patient perspective there are many reasons to question whether a hospital is the best place of care for older adults with frailty. There is some evidence to indicate that hospital care might be potentially harmful to older people due to a lack of mobility that can increase the risk of disability from frailty [2]. The high cost of hospital-based care has also been a major driver to innovate in an economic climate that does not allow for the expansion of hospital bed numbers to match the growth in admission numbers [3]. Avoiding admission to hospital by the provision of hospital-level care in the home is a model of healthcare that is being considered in many countries [4, 5] as hospitals deal with the rise in emergency admissions. However, the scale to which these models of care have transformed health systems is limited, reflecting the lack of evidence to underpin decision-making.

A meta-analysis of randomised trials of admission avoidance hospital at home is limited by the small number of small randomised controlled trials. The evidence suggests that these types of service, that include the option of transfer to hospital, may provide an effective alternative to inpatient care for a select group of older people who require hospital admission [4]. The lack of data on cost-effectiveness also limits the extent to which these types of service form part of a wider policy strategy for acute hospital-level care. It is possible that avoiding admission to hospital reduces the risk of delirium in older people who require hospital-level healthcare. Delirium, a frequent and serious complication in older people who develop an acute illness, is associated with adverse consequences which include increased risk of hospital-acquired complications, new admission to institutional care, new dementia, increased hospital length of stay and increased mortality [6, 7].

### Research objective

The primary objective is to undertake a multi-site randomised trial to estimate the effectiveness of geriatrician-led admission avoidance hospital at home in settings where health and social care provision vary. Secondary objectives are to investigate the incidence of delirium, mortality, new long-term residential care, cognitive impairment, activities of daily living, quality of life and quality-adjusted survival, length of stay, readmission or transfer to hospital, resource use (health and social care and informal care), costs and cost-effectiveness. We will also conduct a process evaluation to describe how the geriatrician-led admission avoidance hospital at home intervention is delivered, how this differs from inpatient care, and how health and social care provision varies between sites; this includes an interview study to explore the experiences of patients and carers.

## Methods/Design

The study protocol was developed using the SPIRIT (Standard Protocol Items: Recommendations for Interventional Trials) Checklist (see Additional file 1).

### Setting and sample

Participants are being recruited from primary care or an acute hospital-based assessment unit in Aneurin Bevan University Health Board; Bradford Teaching Hospitals NHS Foundation Trust; Guy’s and St Thomas’s NHS Foundation Trust, London; Royal Devon and Exeter NHS Foundation Trust; Monklands Hospital, NHS Lanarkshire; St John’s Hospital, NHS Lothian; Southern Health and Social Care Trust, Northern Ireland; Belfast Health and Social Care Trust, Northern Ireland. We are recruiting older people with frailty who require a hospital admission due to an acute change in health. This might be due to acute functional deterioration, delirium, falls and complex comorbidity. There is no simple accepted definition of this population due to variation in the acute presenting illness. However, it is agreed that the degree of prior disability is important and that attempts to define this group should be problem-based [8]. We will describe patients recruited to this trial according to functional dependence, cognitive impairment, comorbidity, history and/or presence of delirium and presenting complaint (such as falls, reduced mobility, confusion, carer strain).

### Eligibility criteria

We are recruiting patients who meet the following eligibility criteria for inclusion: (i) are 65 years and older, (ii) are willing and able to give informed consent for participation in the study, (iii) have been referred to the geriatrician-led admission avoidance hospital at home service and would otherwise require hospital admission for an acute medical event. The presence of a carer will not be a requirement for enrolment and will depend on the individual circumstances of the patient; this will be at the discretion of the clinician responsible for the patient, as is current clinical practice in each centre. Participants will be excluded if they have (i) an acute coronary syndrome (this includes myocardial infarction and unstable angina and is characterised by cardiac chest pain and associated with ECG changes), (ii) require an acute surgical assessment, (iii) have a suspected stroke, (iv) are receiving end of life care as part of a palliative care pathway, (v) refuse admission to the geriatrician-led admission avoidance hospital at home, or are considered by the clinical staff to be too high risk for home-based care, for example those who are physiologically unstable, who are at risk to themselves or if the carer reports hospital at home would not be acceptable, and (vi) patients living in a residential setting.

### Interventions

The intervention group is geriatrician-led co-ordinated, multi-disciplinary healthcare in the home for people who would otherwise be admitted to hospital (otherwise known as admission avoidance hospital at home). Members of the multi-disciplinary team (MDT) include nurses, physiotherapists, occupational therapists and social workers (who might be part of the primary health care team or dedicated staff). The MDT implements treatment and management recommendations, and if required refers to other services (e.g., older peoples’ mental health services, diagnostic services, social workers, dieticians, speech and language therapy, mental health services, pharmacy support and outpatient follow-up). Patients have access to inpatient care, general practitioners and the primary healthcare team. The use of intravenous infusions, administration of medication via a pump and 24-hour care is available in some sites. Healthcare is provided 7 days a week, admissions are restricted to Monday to Friday in all but one site, from 0900 to early evening, and emergency medical cover is available 24 hours a day.

The majority of participants (approximately 80%) who are randomised to inpatient care (the control group) will receive their care by a specialist-led geriatric service. This variation will reflect the challenges of real-life systems and continued pressure on beds. Measures, in the form of participating centres agreeing protocols, have been taken to ensure that the features of usual care are comparable.

### Outcome measures

#### Primary outcome

We are collecting data for each patient on ‘living at home’, defined as the inverse of death or living in a residential care setting, at 6 months follow-up from randomisation.

#### Secondary outcomes

At 6 months follow-up we are measuring:

- Incident and persistent delirium as defined by the Confusion Assessment Method (CAM). The CAM is a brief questionnaire that has been extensively used in research for screening and case ascertainment purposes [9].
- Cognitive impairment with the Montreal Cognitive Assessment (MoCA), the normal range is from 30 to 26 [10].
- Activities of daily living, measured by the Barthel Index [11].
- Mortality
- New long-term residential care
- Length of stay in admission avoidance hospital at home and inpatient admission
- Readmission or transfer to hospital
- Health status (measured by the EuroQoL five dimension (EQ5D) instrument to produce a single index value for use in cost-effectiveness analysis) [12]
- Resource use
- Patient satisfaction using the Patient-Reported Experience questionnaire, developed by Picker Europe and used in the National Audit of Intermediate Care [13].
- We are also collecting data for each patient on ‘living at home’ (the inverse of death or living in a residential care setting) at 12 months follow-up.

### Serious adverse event and adverse event reporting

The potential risks to participants of the research may include a fall (either in the home or inpatient setting), hospital-acquired infection for patients randomised to inpatient admission, hospital admission for those randomised to hospital at home, post-discharge hospitalization and death for all participants. We categorise an adverse event as serious if it results in death, is life-threatening, requires hospitalisation or prolongation of existing inpatient hospitalisation, results in persistent or significant disability or incapacity, or is an important medical event. Expected events for this patient population include falls, pressure sores, hospital or community-acquired infection and transfer to hospital. All serious adverse events (SAEs) which are related to administration of any of the research procedures, and are an unexpected occurrence, either observed by the recruiting clinician or reported by the participant, will be recorded on the case report form (CRF), and forwarded by the site to the trial manager following assessment for seriousness by the site clinician. As a minimum, the following information will be recorded: description, date of onset, end date, assessment of relatedness to the geriatrician-led admission avoidance hospital at home intervention, other attribution/co-intervention and action taken. Follow-up information will be provided as necessary. The chief investigator (CI) or delegate will report SAEs, which in the opinion of one of the clinical leads are ‘related’ and ‘unexpected’ when relating to the study procedures, to the Research Ethics Committee (REC) within 15 working days of the CI becoming aware of the event.

### Recruitment

We have implemented a recruitment pathway that maps to existing arrangements for referral. Eligible participants are identified from patients who are referred by their general practitioner to a single point of access for admission to hospital at home, or who have been transferred from the Accident and Emergency Department to an Acute Assessment Unit and are assessed as suitable for hospital at home. At the point of referral to the trial each participant is provided with a Participant Information Leaflet that describes the research, and they are provided with an opportunity to discuss their questions and concerns about the research with a research nurse. Each participant has the right to withdraw from the study at any time, and if provided the reason for withdrawal will be recorded in the CRF.

### Randomisation procedure and concealment of allocation

The unit of randomisation is the individual participant, who is randomly allocated using a 2:1 ratio (2 admission avoidance hospital at home: 1 inpatient admission) by a local member of the research team who accesses Sortition, the Oxford University’s Primary Care Clinical Trials Unit’s validated in-house online randomisation system. Telephone randomisation is used if sites do not have online access. A computer-generated randomisation sequence is used, and randomisation is stratified by centre, gender and by known cognitive decline [measured by the Informant Questionnaire on Cognitive Decline in the Elderly (IQCODE)] [14]. We have opted for a 2:1 randomisation ratio to deal with the concern expressed by clinical leads that a 1:1 randomisation ratio would place unmanageable pressure on the inpatient services. The success of randomisation is being measured by the number declining to be randomised or withdrawing immediately after randomisation.

### Data collection, management and analysis

**Fig. 1:**
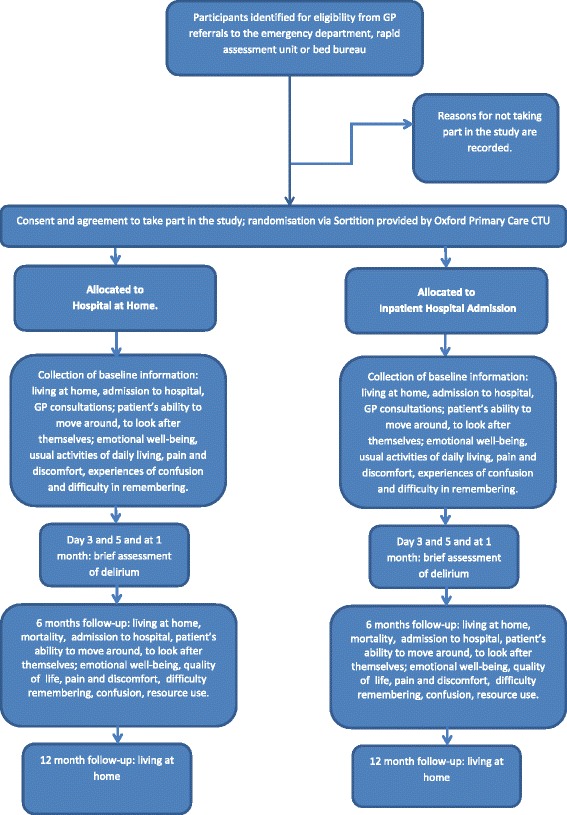
Study flowchart

Research nurses at each site collect data from participants, and from their caregivers if the caregiver is the designated consultee, at baseline and 6 months after randomisation and at 12 months for the primary outcome, with the exception of an assessment of delirium which is at 3 and 5 days, and at 1 month after recruitment (Fig. 1). Each site completes a form to record if death has occurred, with the date; these data are collected from the medical records. Place of residence is recorded by the research nurses at each follow-up visit. Data are collected using a paper form, or directly to an electronic pro forma on Open Clinica.

**Fig. 2:**
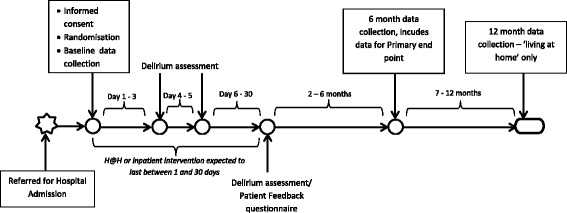
Participant timeline

We collect data at baseline from the patient’s clinical notes and from the clinical lead on the presenting problem that requires admission to hospital, and demographic information (age, education). We also collect data on background cognitive status using the IQCODE, a 16-item informant-based questionnaire, which can also be completed by a carer [14]; incident and persistent delirium measured by the CAM [9], co-morbidity measured by the Charlson Index [15], activities of daily living measured by the Barthel Index [11], current cognitive impairment measured by the MoCA [10], health status measured by the EuroQol five-dimension five-level instrument (EQ-5D-5 L) [12] and major health service use (for example admission to hospital, use of outpatient services and ambulances, home care/help and respite stays in a residential setting) in the 6 months prior to their current illness. If the patient appears to be burdened by the collection of baseline data we use a two-stage approach, with core data (Short IQ Code [14] and Confusion Assessment Method [9]) collected prior to randomisation and following consent, and the remaining data (Barthel Index [11], Charlson Co-Morbidity Index [15], MoCA [10], EQ-5D-5 L [12] and Health Resource Use Questionnaire) collected soon after randomisation (Fig. 2).

At the 6-month follow-up point we collect data from all patients on mortality, new long-term residential care, cognitive impairment, activities of daily living, quality of life, length of stay, readmission or transfer to hospital, and on the number, type and duration of hospitalisations, the use of outpatient and day-case services, the use of community-based health care services including general practitioner consultations and practice-based nursing, medications, respite care, social care including admission to residential or nursing home, and amount of informal care received over the preceding 6 months. Unit costs will be attached to these resource volumes, using National Health Service (NHS) reference costs, Unit Costs of Health and Social Care (PSSRU), and other national data sources. We will collect the costs of the admission avoidance hospital at home intervention in collaboration with providers, and include training time and other elements. This is likely to vary between centres and we aim to collect cost information from all participating centres if possible. At 12 month follow-up we will collect data on living at home (Table 1).

**Table 1:** Case report form

	Study period					
	Baseline	Day 3	Day 5	1 month	6 month	12 month
ENROLMENT						
Eligibility screen	X					
*Informed consent*	X					
*Screening log*	X					
Randomisation	X					
ASSESSMENT						
Assessment form	X	X	X	X	X	X
*Demographics*	X					
*Presenting problem*	X					
*Place of assessment*	X	X	X	X	X	X
*Patient status* (*‘living at home’*)		X	X	X	X	X
*Subsequent admissions*				X	X	
Adverse event log		X	X	X	X	X
QUESTIONNAIRES						
Barthel Index of Activities of Daily Living	X				X	
Confusion Assessment Method (CAM)	X	X	X	X		
Charlson Co-morbidity Index	X				X	
Health Service Use Questionnaire	X				X	
Short Form of the Informant Questionnaire on Cognitive Decline in the Elderly (Short IQCODE)	X					
Montreal Cognitive Assessment (MoCA)	X				X	
EuroQol five-dimension five-level (EQ-5D-5 L)	X				X	
Patient feedback form				X		
Difficulties in Questionnaire completion Form	X	X	X	X	X	
MISCELLANEOUS						
Death form	Completed on patient death					
Discontinuation form	Completed when a patient withdraws or is lost to follow-up					

### Process evaluation

We will complete a structured pro forma to record the key features of the organisation and delivery of admission avoidance hospital at home and of inpatient care, and any local changes to policy and implementation. This will include reviewing care protocols, the method of assessing the patient, and conversations with the staff delivering the intervention. Once recruitment to the trial is established approximately six patients and their carers (until data saturation), from two or more hospital at home and inpatient settings, will be invited to be interviewed at the point of discharge or immediately after discharge from their healthcare setting. Patients and carers will be selected to include those with cognitive impairment, those who are physically frail and who have experienced varied types of health crises (sudden onset of chronic illness, deterioration in the context of multiple health problems and acute exacerbation of a chronic condition) that will impact on the recovery process. The interviews will assess the process of care and how the healthcare they received facilitated recovery, as well as relatives/caregivers perceptions and experience of HAH and inpatient care. Interviews will be recorded. We will also complete a structured pro forma to record the key features of the HAH with CGA intervention and of inpatient care, this will include the use of care protocols, the method of assessing the patient, and conversations with the staff delivering the intervention.

### Data management

We store all paper and electronic data in a secure environment, and refer to the participant by a trial number and not by name, except for the signed consent form. We follow the standard operating procedures of the University of Oxford Primary Care Clinical Trials Unit, which are fully compliant with the Data Protection Act and Good Clinical Practice (GCP). No-one outside the study team has access to either the CRFs or the database; members of the research team can access patient identifiable data in order to collect follow-up data. Direct access will be granted to authorised representatives from the sponsor and host institution for monitoring and/or audit of the study to ensure compliance with regulations. Staff at each site enters data directly to a secure laptop or computer or on paper versions of the CRF and questionnaires. Data from paper forms are double entered. Researchers from each site are asked to check data for completeness prior to returning, for those sites entering data directly into the database internal validation checks are run for each data field. Any inconsistencies and missing data are highlighted as queries which are then returned to the site to be resolved. With regards paper CRFs, the same checks are applied once the data managers have performed the double data entry. Data queries are returned to sites for resolution on a regular basis. Interviews will be audio recorded, fully transcribed and entered into NVivo software. Field notes pertaining to the physical, social and care environment of the home setting will also be managed and analysed. All audio recordings and field notes will be stored safely in confidential conditions and electronic data in a secure, protected environment.

#### Study oversight

The overall supervision of the trial is carried out by the Trial Steering Committee. An independent Data Monitoring Committee meets every 6 months to review trial progress and data, this includes all SAE reports. The sponsor is the University of Oxford.

### Statistical methods

#### Sample size

We calculated the sample size for the primary outcome ‘living at home’ (the inverse of death or living in a residential care setting) at 6 months follow-up, for a 2:1 randomisation ratio with two-thirds randomised to admission avoidance hospital at home and one-third to inpatient admission. Several sources informed our estimate of effect size, these included an audit of 750 patients who received admission avoidance hospital at home in Lanarkshire, and the summary estimate from a Cochrane Review of admission avoidance hospital at home that was published in 2008 [16]. Our proposed study effect estimate is based on a control group (inpatient admission) event rate at 12 months of 50% living in a residential setting, with a 10% reduction to 40% in the admission avoidance hospital at home group, equal to a relative risk of 0.8 which lies towards the top end of the 95% confidence interval (CI) for the pooled estimate reported in the Cochrane Review [16]. We have calculated that to achieve 90% power at a significance level of 0.05, we will need to recruit 1350 participants to detect a 10% absolute difference. The estimated recruitment rate allows 15% attrition resulting in a projected sample size of 1552. We have re-examined the sample size calculation using an estimate of the intra-cluster correlation (ICC) of 0.005, this would provide 88% power to detect the assumed effect size of RR = 80%, for a two-tailed alpha of 0.05.

### Analysis

We will analyse the data using an intention-to-treat analysis and based on a mixed effect model adjusting for recruitment centre (random effects) and individual patient characteristics. For the primary outcome of living at home 6 months, the primary analysis we will use a mixed-effect logistic regression model including data available on all randomised patients at 6 months and 12 months follow-up, adjusting for recruitment centre, gender, and IQCODE score at baseline. In the model, participants and centres will be fitted as random effect, and time and treatment as fixed effect. We will fit an interaction term between time and intervention group so that possible differences of intervention effect could be assessed at each time point. Although, the method has the advantage of implicitly accounting for the data missing at random mechanism, we will also assess the impact of missing data on the primary analysis by carrying out sensitivity analyses based on imputing (multiple imputation) the missing values. We will use similar models for all binary outcomes (presence of delirium, cognitive impairment, etc.), and will use equivalent models for continuous outcomes (e.g. normal distribution). We expect length of stay to be highly skewed so other parametric models might be required for this, and in the case of poor fit we will use simple non-parametric tests (non-adjusted) for this outcome. We have planned one subgroup analysis of the effect of care setting (home versus hospital) on the incidence of delirium in people who are cognitively impaired (defined by the MoCA) [10].

#### Economic analysis

We will calculate the costs in each arm of the study on an intention-to-treat basis, and will report these from a health and social care perspective; in addition we will quantify and value the cost of informal care. We will calculate quality-adjusted life years from the responses to the EQ-5D using the UK “tariff”, and use linear interpolation between baseline and 6-month values and adjusting for within-trial mortality. We will collect resource-use information on health and social care services used, including the organisation and delivery of the interventions, hospital inpatient stays and procedures, outpatient and day-case use, hospital at home durations, other consultations (including general practitioner and community nurse consultations), medications, adverse events, admission to respite care and long-term care and use of other social care. Resource use volumes will be multiplied by appropriate national unit costs such as NHS Reference Costs to derive a cost per participant. We will use an average cost in the main analysis, and information on variability will be used in sensitivity analyses and to explore the possible costs of generalising the service nationally if proven to be cost-effective. We will estimate the main cost-effectiveness measure as the net cost per quality-adjusted life year gained, for the within-trial period. In the event of a within-trial difference in mortality, we will estimate average life expectancy using information from life tables and relevant cohort studies, and estimate quality-adjusted life years gained/lost. We will handle uncertainty concerning the reported cost-effectiveness ratio using the non-parametric bootstrap and will report cost-effectiveness acceptability curves, scatters on the cost-effectiveness plane, and 95% confidence intervals (using the percentile method) around the net benefit statistic. We will conduct a sensitivity/scenario analyses for different cost perspectives, and these will include analyses in which the costs of admission avoidance hospital at home and inpatient care are allowed to vary across a range observed in the study, and analyses in which informal care costs are excluded or included.

#### Analysis of the key features of contextual data

A narrative, descriptive account of the organization of services at each centre will be produced drawing on data collected from individual sites to populate a structured pro forma; this will include an events log (that will include discussions with staff) and formal documents relating to organisation and delivery. We will focus on the dimensions of the different settings and services, and any changes to staffing and service organisation that might impact on the delivery of healthcare (e.g., a reduction in geriatrician input; ward re-organisation/closures; expansion/contraction of the scope of admission avoidance hospital at home). We will compare sites and services, and develop hypotheses to explore patterns of variation in trial outcomes.

#### Analysis of qualitative interviews with patients and caregivers

We will use a grounded theory analytic approach in the qualitative study, combining simultaneous data collection and analysis, constant comparison and search for negative cases. The rationale for the adoption of a grounded theory approach to analysis is twofold. First, the approach is flexible yet systematic and robust through the use of iterative, simultaneous data collection and analysis, constant comparison, search for native cases and memo writing to generate concepts and categories as well as their properties and dimensions through the coding process. A second important feature is a focus on context and process. This approach will provide a more robust, systematic and in-depth approach to addressing issues of context and process, critical in this study. Our coding process will generate elements that can be grouped into concepts and then into higher order categories, which will form the basis of our theory of patients’ perception of recovery and the factors contributing to it (e.g., personal and social resources, content and process of service delivery). We will recruit additional respondents based on key features to test out aspects of our developing grounded theory (via theoretical sampling).

## Discussion

The research outlined in this protocol is of increasing importance given the current focus of policy in a number of countries, including the UK, on providing coordinated care for older people (including those with cognitive impairment) that is closer to home. Furthermore, if a benefit of a reduced incidence of delirium is confirmed by the results of this randomised controlled trial there will be significant implications for vulnerable patients with reduced functional and cognitive decline.

### Trial status

Recruitment is ongoing

## Additional file

Additional file 1: Standard Protocol Items Recommendations for Interventional Trials (SPIRIT) 2013 checklist recommended items to address in a clinical trial protocol and related documents. (DOC 121 kb)
